# Entomocidal effects of beech apricot, *Labramia bojeri,* seed extract on a soybean pest, the velvetbean moth, *Anticarsia gemmatalis,* and its enzymatic activity

**DOI:** 10.1093/jis/14.1.27

**Published:** 2014-01-01

**Authors:** Maria L. R. Macedo, Carlos E. G. Kubo, Maria G. M. Freire, Roberto T. A. Júnior, José R. P. Parra

**Affiliations:** 1 Laboratório de Purificação de Proteínas e suas Funções Biológicas, Depto. De Tecnologia de Alimentos e Saúde Pública, Naturais, Universidade Federal de Mato Grosso do Sul, CP 549, CEP 79070-900, Campo Grande, MS, Brazil; 2 Laboratório de Química e Biomoléculas, Centro de Pesquisas, ISECENSA, Campos dos Goytacazes-RJ, Brazil; 3 USP/ESALQ –Departamento de Entomologia e Acarologia, CP 09 – 13418-900, Piracicaba, SP, Brazil; 3 Universidade de São Paulo, Escola Superior de Agricultura Luiz de Queiroz, Departamento de Entomologia. Av. Pádua Dias, 11, 13418-900 -Piracicaba, SP, Brasil

**Keywords:** insecticidal, larval survival, velvetbean, weight reduction, nutritional index, action mechanism

## Abstract

The effects of the beech apricot, *Labramia bojeri* A. de Candolle (Sapotales: Sapotaceae), seed aqueous extract on the larval development of the velvetbean moth, *Anticarsia gemmatalis* Hübner (Lepidoptera: Noctuidae), was evaluated. The extract inhibited larval development, pupal weight, and survival and emergence of adults. Digestive proteolytic activity in larval midgut and feces extracts was determined. Larvae fed 10 g/L of the aqueous extract showed a significant reduction in trypsin activity (~64%), when compared with control larvae. Trypsin and chymotrypsin activities were also detected in fecal material in aqueous-extract-fed larvae, with about ~4.5 times more trypsin activity than the controls. The results from dietary utilization experiments with *A. gemmatalis* larvae showed a reduction in the efficiency of conversion of ingested food and digested food and an increase in approximate digestibility and metabolic cost. The effect of the extract suggests the potential use of *L. bojeri* seeds to inhibit the development of *A. gemmatalis* via oral exposure. The *L. bojeri* extract can be an alternative to other methods of control.

## Introduction

The velvetbean moth, *Anticarsia gemmatalis* Hübner (Lepidoptera: Noctuidae), is one of the main pests of soybean, *Glycine max* L. (Merrill) (Fabales: Fabaece), a legume that provides about half the global demand for vegetable oils and proteins. *A. gemmatalis* causes extremely high levels of defoliation when infestation is heavy and can severely damage axillary meristems ( Walker et al. 2000 ; Oerke 2006 ).

For the control of pests in storage areas, methyl bromide (MeBr) and phosphine (PH3) are used, but they may cause several problems in stored products ( Khani et al. 2011 ). In addition, their widespread use has led to some serious problems, including the development of insect strains resistant to insecticides ( Damásio et al. 2007 ; Garriga and Caballero 2011 ).

The deleterious effects of plant extracts on insects are manifested in several ways, including toxicity, growth retardation, feeding inhibition, oviposition, repellence, suppression of calling behavior, and reduction of fecundity and fertility ( Muthukrishnan and Pushpalatha 2001 ; Baskar et al. 2009 ). Therefore, the objective of this study was evaluate the bioefficacy of an aqueous extract of beech apricot, *Labramia bojeri* A. de Candolle (Sa-potales: Sapotaceae), on *A. gemmatalis* development, nutritional index, digestive proteinase activity, and zymogram analyses of the digestive proteinase activities. In Brazil, it is common to find *L. bojeri* in all regions. The results of this study suggest that small farmers could collect these seeds, make an aquous extract, and use it for control.

## Materials and Methods

### Plant material

*Labramia bojeri* seeds were collected in the city of São João da Barra, in the State of Rio de Janeiro, Brazil.

### Insects

The eggs of *A. gemmatalis* were obtained from Dr. J. R. P. Parra (Laboratório de Biologia dos insetos, ESALQ-USP, Piracicaba, SP, Brazil), and a culture was maintained in the Laboratório de Purificação de Proteínas e suas Funções Biológicas, Universidade Federal de Mato Grosso do Sul, Campo Grande, Brazil.

### Aqueous extract

*Labramia bojeri* seeds were triturated, and the finely ground product was mixed with distilled water at concentrations of 10 and 20 g/L. The suspension was kept for 24 hr at room temperature to extract the soluble compounds. The mixture was then filtered through fine fabric (voile), and the aqueous extract was stored at 4º C. A new extract was made every three days and used within two days.

### Effects of an aqueous extract on the development of *Anticarsia gemmatalis*

Soybean leaves (genotype IAC-19) from the middle third of plants at the R1 and R2 stages (beginning and full bloom, respectively), according to Fehr and Caviness (1977) were immersed for 2 sec in aqueous extract or distilled water (control) before being offered to larvae (up to the 4 ^th^ stadium). After evaporation of excess water, humidified cotton was placed around petioles to maintain leaf turgor. For each treatment, five neonate larvae were placed in a Petri dish (15.0 x 1.0 cm) lined with paper filter. Each treatment was repeated 20 times for each of the above concentrations (n = 100). Larvae fed on a diet containing 10 g/L *L. bojeri* aqueous extract (LbAE) were used to analyze other biological parameters, such as larval growth, pupal weight, development and mortality, adult longevity, and produced malformations in pupae and adult insects.

### Nutritional Parameters

A number of nutritional parameters were compared among 4 ^th^ instar larvae exposed to either the LbAE-treated or control diet. The larvae, feces, and remaining uneaten food were separated using a microscope, dried, and weighed. Nutritional indices of consumption, digestion, and utilization of food were calculated, as described by Waldbauer (1968) and Farrar et al. (1989) . The nutritional indices, namely efficiency of conversion of ingested food (ECI), efficiency of conversion of digested food (ECD), and approximate digestibility (AD) were calculated as follows:

$\text{ECI} = (\ddot{\text{A}}\text{B/I})\times 100$

$\text{ECI} = [(\ddot{\text{A}}\text{B/I}\times \text{F})]\times 100$

$\text{AD} = [(I\times F)/I]\times 100$

where I = weight of food consumed, ÄB is the change in body weight, and F = weight of feces produced during the feeding period. Metabolic cost (MC) was calculated as:

$\text{MC} = 100 - \text{ECD}$

### Midgut preparation

Proteinases were obtained from the midguts of 4 ^th^ instar larvae according to Macedo et al. (1995) . Fourth instar larvae were cold-immobilized, and the midgut, along with its contents, was removed in cold 150 mM NaCl and stored frozen (-20º C) until needed. Guts from the larvae were subsequently homogenized in 150 mM NaCl and centrifuged at 6,000 x g for 5 min, and the supernatants were pooled and kept on ice for enzymatic assays. The protein concentration of the extracts was determined according to Bradford (1976) .

### Fecal pellet preparations

Proteinases were obtained from the midguts of 4 ^th^ instar larvae according to Macedo et al. (2010) . Feces of the caterpillars were collected during the experiment and frozen (-20° C). When necessary, feces were macerated, homogenized in 200 mM Tris-HCl buffer (Tris – hydroxymethyl aminomethane), pH 8.5, and centrifuged at 20,000 g for 30 min at 4º C, and supernatants were used for *in vitro* enzymatic assays.

### Enzymatic assays

The proteolytic activity was estimated using chromogenic substrates such as BApNA (N-benzoyl-DL-arginyl-p-nitroanilide) and SAAPFpNA ( *N-* succinyl-alanine-alanine-proline-phenylalanine p-nitroanilide) at a final concentration of 1 mM. Midgut and feces larvae extracts (50 µg protein) were incubated in 100 mM Tris-HCl buffer, pH 8.0, in a final volume of 0.1 mL for 10 min before the addition of substrate. The reactions were allowed to proceed at 37º C for 20 min and then stopped by adding 0.2 mL of 30% (v/v) acetic acid. The results of *in vitro* enzyme analyses are reported as the means of three independent experiments with appropriate blanks, and absorbance was measured at 410 nm.

### Proteinase activity of extracts in polyacrylamide gel containing 0.1% gelatin

Proteins extracted from the midguts and fecal extracts of larvae fed on diets with or without LbAE, and without prior boiling or reduction, were run on SDS-PAGE (10% gels) with some alterations. Midgut and fecal extract proteins from the larvae fed with an artificial diet and fed with 10 g/L LbAE were incubated with 100 µM TLCK (a specific inhibitor of trypsin) for 10 min at 30° C. These mixtures were subjected to SDS-PAGE in gels containing 0.1% gelatin. Following electrophoresis at 5° C, the gels were washed with 2.5% Triton X-100 solution (Sigma-Aldrich, www.sigmaaldrich.com ) for 2 hr with shaking to remove the SDS, after which the gels were incubated with 0.1 M Tris–HCl, pH 8.0, for 2–3 hr. The gels were subsequently stained with Coomassie Brilliant Blue R-250.

### Statistical analysis

All data were examined using the Mann-Whitney test. The *p* < 0.05 level was considered to be significant.

## Results

### Effect of LbAE on fourthinstar larvae

**Figure 1.**
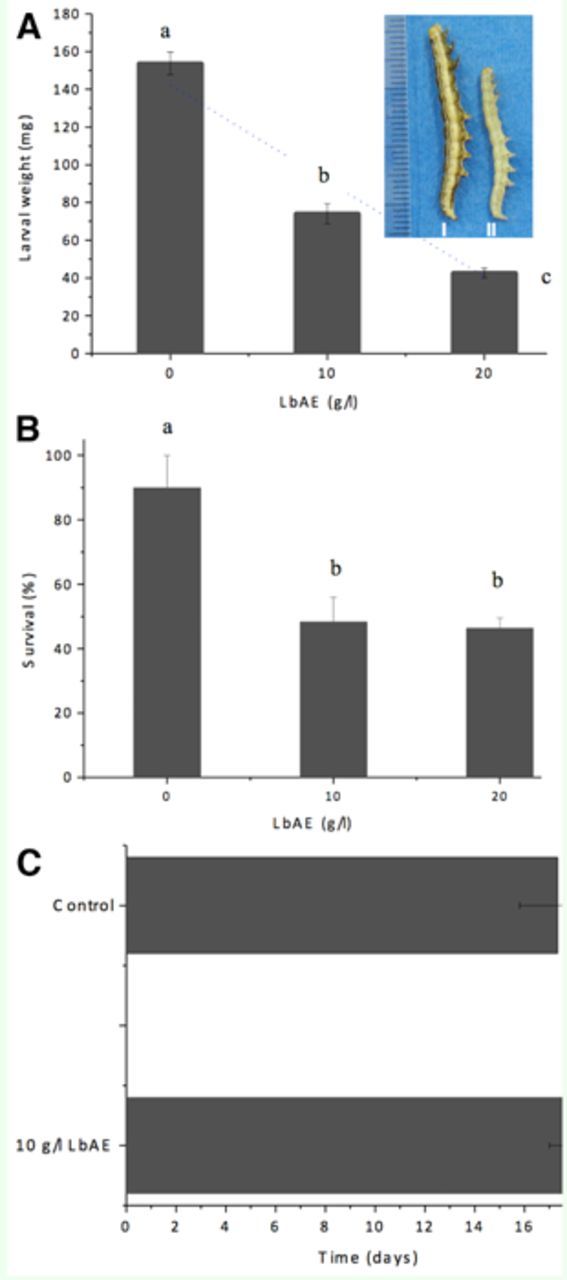
Effect of dietary LbAE on *Anticarsia gemmatalis* larval mass (A) and survival (B). Effects of 10 g/L LbAE on larval development time (C). Insert: variation in the size of 4th instar *A. gemmatalis* larvae fed 10 g/L LbAE (right) and control (left) diets. Ruler = cm. Bars indicate standard error of the mean. The same letters indicate that there were no significant statistical differences (Mann-Whitney U test, n = 50, *p =* 0.05). High quality figures are available online.

Larvae fed on diet containing 10 g/L LbAE produced an approximate 50% decrease in weight ( Figure 1A ). LbAE of up to 20 g/L caused ~ 75% decrease in larval weight ( Figure 1A ). Treatment containing aqueous extract at 10 g/L and 20 g/L reduced survival by about 50% ( Figure 1B ). However, 10 g/L LbAE did not significantly affect larval development time ( Figure 1C ).

### Effects of LbAE on development stages

**Figure 2.**
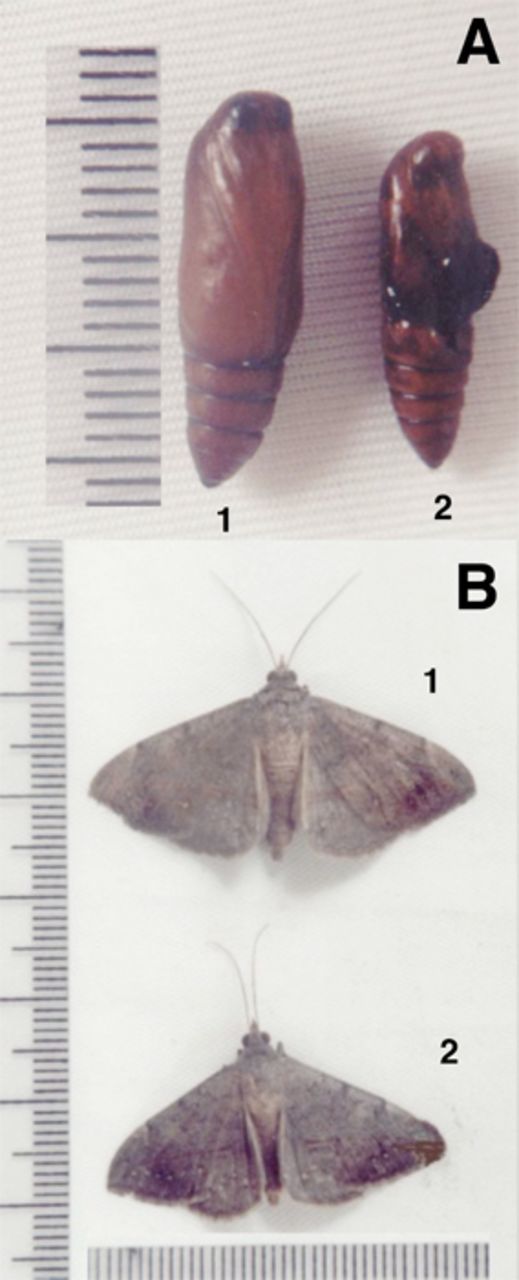
Effect of LbAE on the formation of pupae and adult *Anticarsia gemmatalis* . (A) Pupa: lane 1, control-fed larva; lane 2, LbAE-fed larvae. (B) Adult insects: lane 1, control-fed larva; lane 2, LbAE-fed larva. High quality figures are available online.

Pupal viability was significantly reduced by ~ 35% for larvae fed on a diet supplemented with 10 g/L LbAE ( Table 1 ). Although there was no difference in the duration of pupation, at 24 hr after pupation, the weight of larvae that were fed on the LbAE-containing diet was significantly lower (~ 18 %) than that of larvae fed on the control diet. Pupal malformation (25%) was also observed, with the pupae of the larvae that were fed with LbAE showing abdominal and thoracic deformations and being smaller in size than the larvae that were fed the control diet ( Figure 2 A; Table 1 ).

**Table 1.**
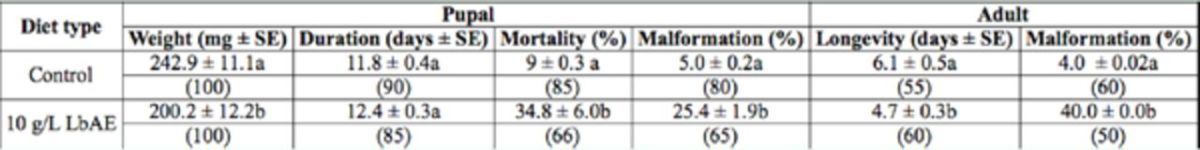
Effect of *Labramia bojeir* aquous extract (LbAE) on survival and growth of *Anticarsia gemmatalis* . Sample sizes are indicated in parentheses under the mean.

Means followed by the same letter within each column are not significantly different ( *p* > 0.05; Mann-Whitney U test).

The viability of adult moths was evaluated by counting the number of malformed moths at emergence and by determining their longevity. Adult insects from larvae fed with LbAE were significantly smaller in size by 40% ( Figure 2 B; Table 1 ) than those from larvae fed on the control diet. The longevity of adult insects developed from the larvae fed on the control diet was about 1.4 days longer than that of adults from the larvae fed with LbAE ( Table 1 ).

### Nutritional parameters

**Figure 3.**
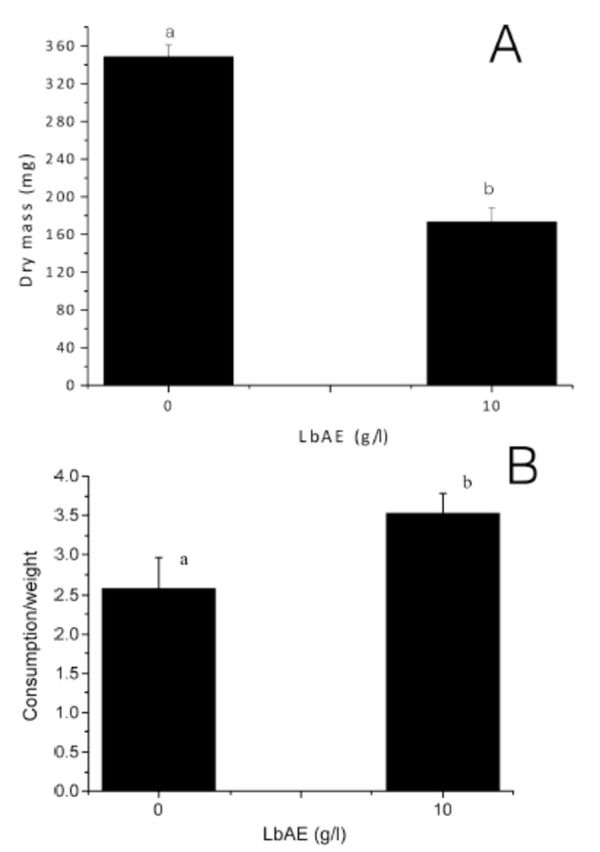
Physiological parameters measured for *Anticarsia gemmatalis* larvae. The larvae were fed on a control diet or diets containing LbAE for 16 days. (A) Diet consumption by larvae (in mg, based on dry mass). (B) Mean food consumption as a ratio of the mean larval weight. Bars indicate standard error of the mean. The same letters indicate that there were no significant statistical differences (Mann-Whitney U test, n = 50, *p =* 0.05). High quality figures are available online.

The larvae reared on an LbAE-containing diet consumed ~ 47% less food than the controls ( Figure 3 A). However, when food consumption was expressed as a ratio of body weight, the larvae fed with LbAE consumed 25% more than the controls ( Figure 3 B). LbAE had a toxic effect when ingested by larvae ( Table 2 ). LbAE, when incorporated in an artificial diet at 10 g/L, reduced ECI and ECD and increased the MC and AD for larvae when compared with the control.

**Table 2.**
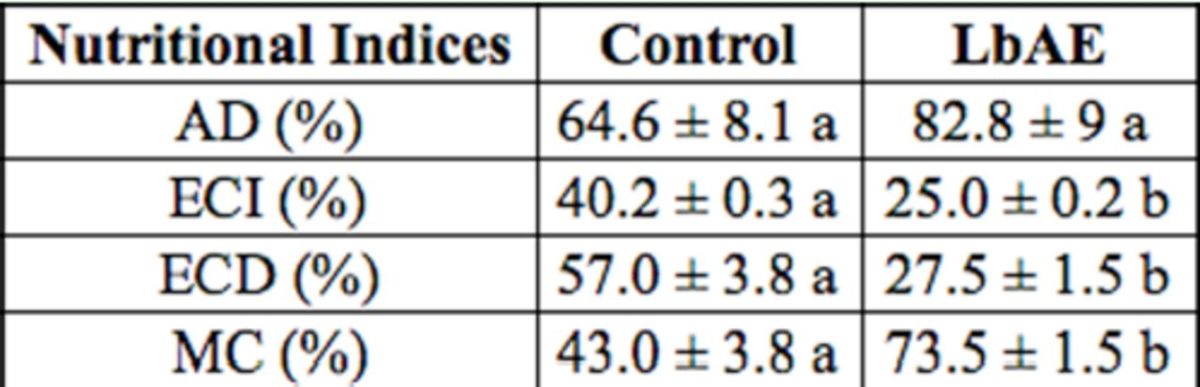
Nutritional indices of *Anticarsia gemmatalis* fourthinstar larvae on *Labramia bojeir* aquous extract (LbAE) and control diet.

Means followed by the same letter within each row are not significantly different ( *p* > 0.05; Mann-Whitney U test). AD = approximate digestibility; ECI = efficiency of conversion of ingested food; ECD = efficiency of conversion of digested food; MC = metabolic cost.

### Enzymatic activities

**Figure 4.**
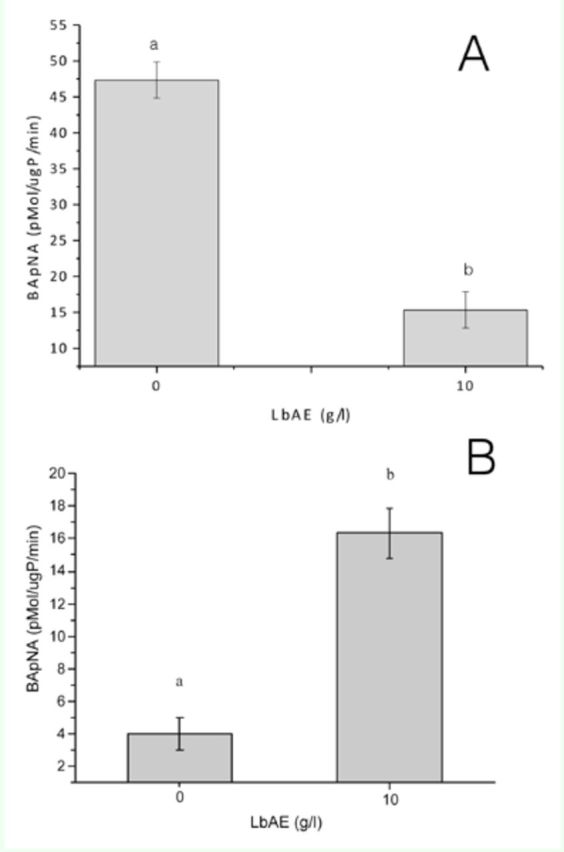
Midgut trypsin-like activities in *Anticarsia gemmatalis* larvae after exposure to LbAE. Trypsin activity in midguts (A) and fecal material (B). Error bars indicate standard error of the mean. There was a significant difference between LbAE and control larvae in all treatments (ANOVA, *p* < 0.05). High quality figures are available online

Extracts of soluble proteins prepared from midguts contained enzymes capable of hydrolyzing the synthetic substrates BApNA and SAAPFpNA, but were unable to hydrolyze BTpNA (data not shown). 10 g/L LbAE-fed larvae showed a significant reduction in trypsin activity (~ 64%) when compared with control larvae ( Figure 4 A). In contrast to the trypsin activity, there was no significant change in the midgut chymotrypsin activity of LbAE-fed larvae (data not shown). Typsin and chymotrypsin activities were also detected in fecal material, with LbAE-fed larvae presenting ~ 4.5 times more trypsin activity than the controls ( Figure 4 B), whereas there was no alteration in the chymotrypsin activity of these larvae (data not shown).

### Polyacrylamide gels containing 0.1% gelatin

**Figure 5.**
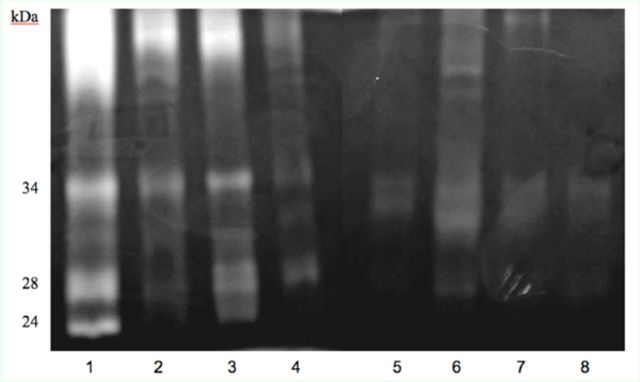
SDS-PAGE of midgut and feces extracts of 4th instar *Anticarsia gemmatalis* larvae. (1) Larvae midgut extract of control treatment; (2) Larval midgut extract of aqueous extract treatment at 10g/L; (3) Larvae midgut extract of control treatment with TLCK; (4) Larvae midgut extract of 10g/L aqueous extract treatment with TLCK; (5) Extract of larvae feces following control treatment; (6) Extract of larvae feces following 10g/L aqueous extract treatment; (7) Extract of larvae feces following control treatment with TLCK; (8) Extract of larvae feces following 10g/L aqueous extract treatment with TLCK. High quality figures are available online.

Polyacrylamide gels containing 0.1% gelatin were used to examine the action of LbAE on trypsin activity and to analyze the profile of these enzymes. Figure 5 shows a decrease in the trypsin activity of midgut extracts of LbAE-fed larvae when compared with that of the control larvae. The band of approximately 24 kDa had its tryptic activity strongly reduced. There was an increase in the trypsin activity of fecal extracts of LbAE-fed larvae when compared to that of the control larvae.

## Discussion

The search for alternative ways of controlling agricultural insect pests has led to the investigation of naturally-occurring compounds from plants that may have toxic, repellant, antifeedant, or anti-hormonal properties ( Nathan et al. 2007 ). LbAE significantly affected larval development, pupal weight, and mortality, and caused malformations. LbAE reduced the survival and longevity of adult moths and produced malformations in 25% of the individuals examined, important parameters with respect to insect control ( Nathan et al. 2007 ). Larvae reared on an LbAE-treated diet consumed 47% less than larvae on the control diet. However, when the consumption was expressed as a ratio of body weight, the LbAE-fed larvae consumed 25% more than the controls. Thus, LbAE apparently did not affect the feeding pattern, as has been previously observed for *A. gemmatalis* ( Macedo et al. 2010 ). The reduction in larval weight, despite the increase in consumption for LbAE-fed larvae, suggests that LbAE inhibits nutrient uptake in *A. gemmatalis* ( Eisemann et al. 1994 ).

An index of dietary utilization showed that ECI and ECD decreased when larvae were fed the LbAE diet. In the present study, the AD value for larvae of *A. gemmatalis* was increased throughout the feeding period of the experiment. AD is also evaluated as a function of the time that food is retained in the digestive tract. Thus, an increase in AD leads to a decrease in the ECI. This finding suggests that, during this treatment, the food remained for a greater time in the insect’s gut, allowing the detoxification of the protein. A greater AD would help to meet the increased demand for nutrients and compensate for the deficiency in food conversion (reduction in ECI and ECD), perhaps by diverting energy from biomass production into detoxification. This behavior has also been observed by others ( Coelho et al. 2007 ; Ramos et al. 2009 ). A drop in ECI indicates that more food is being metabolized for energy and less is being converted to body mass, i.e., growth of the insect ( Koul et al. 2003 ). ECD also decreases as the proportion of digested food metabolizes for energy increases. Confirming these results, Table 2 demonstrates an increase of ~ 80% in MC in *A. gemmatalis* larvae. The reduction in ECD is likely the result of a reduction in the efficiency of converting food into growth, perhaps by a diversion of energy from production of biomass into detoxification of LbAE, i.e., an increase in metabolic costs. The results obtained in our study are in agreement with those obtained by Coelho et al. 2007 and Ramos et al. 2009.

Enzymatic assays were carried out to examine the mechanisms of LbAE toxic action. Trypsin-activity in the midgut was decreased in the larvae that fed on leaves treated with an aqueous extract at 10 g/L. Elevated levels of trypsin activity were also observed in the fecal material of LbAE-fed larvae. A change in the membrane environment and consequent disruption of enzyme recycling mechanisms may provide an explanation for the observed increases in the tryptic activity of fecal extracts collected from both GNA and Con A fed larvae ( Fitches and Gatehouse 1998 ). The increase in the trypsin activity of feces from LbAE-fed larvae suggests that LbAE may cause the rupture of the peritrophic membrane of *A. gemmatalis* ( Ramos et al. 2009 ). A polyacrylamide gel containing 0.1% gelatin corroborated these results, where samples of extracts of larvae midgut and feces with and without TLCK confirmed major trypsin-activity and differences between the treatments. No novel enzymes were induced in larvae reared on a diet containing LbAE.

The effects of LbAE on soluble trypsin activity suggest that LbAE significantly decreases trypsin levels in the larval gut and affects the recycling mechanisms of this enzyme, since LbAE increases trypsin activity in the feces more than in the gut ( Fitches and Gatehouse 1998 ). These differential trypsin activities can lead to poor nutrient use, retarded development, and eventually death by starvation ( Gatehouse et al. 1979 ; Macedo et al. 2004b ), as midguts of larval Lepidoptera contain mainly serine proteinases ( Terra and Ferreira 1994 ; Abdeen et al. 2005 ).

The results presented in this study provide further evidence for the potential use of this LbAE for inhibition of the development of *A. gemmatalis* via oral exposure. The use of complex mixtures as pest control agents is advantageous because natural mixtures may act synergistically ( Grassi et al. 2005 ) and may present greater overall bioactivity compared to the individual constituents ( Chen et al. 1995 ).

## Abbreviations

AD: approximate digestibility
ECD: efficiency of conversion of digested food
ECI: efficiency of conversion of ingested food
LbAE: L. bojeri seed aqueous extract
MC: metabolic cost
